# Stakeholder engagement to identify barriers to implementation and inform the development of point-of-care diagnostics for TB

**DOI:** 10.5588/ijtldopen.25.0680

**Published:** 2026-04-13

**Authors:** I. Salles, E. Lessem, L. Walshe, B. Myrzaliev, N.B. Hoa, M. Shah, R.E. Chaisson, Y.C. Manabe

**Affiliations:** 1 Division of Infectious Diseases, Johns Hopkins University School of Medicine, Baltimore, MD, USA;; 2 Department of International Health, Johns Hopkins Bloomberg School of Public Health, Baltimore, MD, USA;; 3 KNCV TB Foundation, Branch Office KNCV in Kyrgyzstan, Bishkek, Kyrgyzstan;; 4 Vietnam National Lung Hospital, Vietnam National Tuberculosis Program, Hanoi, Vietnam;; 5 Hanoi Medical University, Hanoi, Vietnam.

**Keywords:** tuberculosis, stakeholder engagement, health care delivery, implementation barriers, training gaps, capacity building

## Abstract

**BACKGROUND:**

Rapid TB diagnosis is crucial for improving outcomes and reducing transmission, yet nearly one third of cases remain undiagnosed or unreported. Point-of-care (POC) diagnostics are critical for bridging this gap.

**METHODS:**

We surveyed stakeholder priorities for TB POC diagnostics to inform development aligned with global needs. An online survey was disseminated through SMART4TB partner networks over 13 weeks. The survey captured preferences for diagnostic features, testing obstacles, and attributes to strengthen TB response. Ranked preferences were analysed using weighted scoring with subgroup comparisons.

**RESULTS:**

Of 274 respondents, 89% were from high-burden countries and represented diverse stakeholders; 57% (155/274) were TB care providers. Accuracy and rapid turnaround time to results were top diagnostic features. Primary obstacles were external sample transport and consumables availability. Key attributes to effective TB response included sensitive POC tests, close follow-up, and more personnel training to recognise TB. Paediatric TB screening training was infrequent, with 53% rarely or never trained.

**CONCLUSION:**

Accuracy and rapid turnaround are priority attributes for new TB POC diagnostics. Overcoming systemic barriers, including robust systems for identifying those who need screening, sample transport, material availability, and care continuity, is critical to achieving the successful implementation, and full impact of POC diagnostics.

TB remains the leading infectious cause of death globally, overtaking COVID-19 in 2024.^1^ Almost one third of 10 million people living with TB annually remain undiagnosed, perpetuating the ‘diagnostic gap’.^2^ Accurate, easy-to-use, point-of-care (POC) diagnostics that deliver same-day results at the point of need may close this gap by facilitating timely treatment and reducing pre-treatment loss.^3^ Even in well-resourced settings, patients experience diagnostic delays, underscoring the need for accessible and accurate POC diagnostics.^4^

The WHO End TB Strategy established targets to reduce incidence by 90% and mortality by 95% by 2035.^1^ Development and implementation of new tools to improve TB detection and treatment are necessary to achieve these goals. WHO Target Product Profiles (TPPs) were developed with technical experts and academic researchers to outline ideal TB POC diagnostic characteristics including diagnostics that use easily obtainable specimens, deliver accurate results within a single visit, and are affordable and accessible.^2^ Expanding such tools could strengthen TB control in resource-limited settings^5^ by enabling earlier diagnosis and care, thereby reducing transmission and mortality.^6^ A TPP priority is deployment of improved POC diagnostics at the most decentralised levels of health systems^2^ where most people first seek care, such as primary and community-based health facilities. These facilities often lack onsite diagnostic capacity, requiring referrals to higher-level centres^2,7^ and contributing to attrition in the TB care continuum.^8,9^

Uptake of current WHO-recommended rapid diagnostics is limited: in 2023, only 48% of individuals newly diagnosed with TB received a rapid molecular test, well below the global target of 100%.^1^ Closing the diagnostic gap requires new tools that reach individuals currently missed by TB services. It is therefore essential to align diagnostic development with needs and preferences of end-users and affected communities. Incorporating perspectives of people affected by TB can enhance effectiveness of existing health services and the impactful use of innovations like improved TB POC diagnostics.^10,11^

Our objective was to gather diverse international stakeholder perspectives on priorities and preferences for novel TB POC diagnostics and to identify barriers to implementation to inform test development. This study was conducted as part of the SMART4TB (Supporting, Mobilizing, and Accelerating Research for Tuberculosis Elimination) Consortium.

## METHODS

A cross-sectional survey was conducted between 19 May and 22 August 2024, among 274 individuals engaged in TB care, treatment, or support. Participants were identified through the SMART4TB global consortium database of professionals in TB research, clinical care, policy, and community engagement. Recruitment communications encouraged dissemination through partner networks to enhance geographic and stakeholder representation. Eligible participants were adults (≥18 years) who had experienced TB or were involved in TB care, treatment, or support. The survey included multiple-choice, ranking, and open-ended questions across six domains: 1) demographics and professional background; 2) facility diagnostic capacity; 3) prioritisation of POC diagnostic attributes; 4) obstacles to TB testing; 5) attributes to strengthen TB response; and 6) acceptable turnaround time and cost. To ensure relevance and minimise burden, skip logic directed participants to role-specific question blocks, resulting in variable response counts; participants could exit at any time.

Participants ranked three most- and least-important POC diagnostic features and selected three considered beneficial but not essential. They also ranked top three obstacles to TB testing and attributes to strengthen TB response. The feature list was developed from literature and refined with expert input.

A weighted scoring system was applied. For POC diagnostic features, weights were 4 (most important), 3 (second), 2 (third), 1 (beneficial but not essential), −1 (third least important), −2 (second least), and −3 (least). For obstacles to TB testing and attributes to strengthen TB response, only positive weights were applied: 3 (most important), 2 (second), and 1 (third). Weighting schemes reflected the design of ranking tasks: the first incorporated both beneficial and least-important selections, while the second captured only most-important selections. Weighted scores were summed across rank categories and normalised by dividing by number of respondents, yielding a composite preference score for each item. Higher scores indicated greater prioritisation, while lower scores indicated lower prioritisation. Subgroup analyses assessed variation by stakeholder role. Additional subgroup analyses examined whether priorities differed by TB burden setting and direct involvement in TB evaluation (Supplementary Data Figures S1–S6).

Data were collected via the Qualtrics platform, providing a secure, encrypted environment. Descriptive statistics (means, medians, standard deviations, proportions) were calculated in Stata 18SE. All participants provided electronic informed consent. Ethical approval was granted by Johns Hopkins Medicine Institutional Review Board (IRB00401794).

## RESULTS

A total of 274 individuals participated in the survey, with broad global representation; 89% provided TB care, treatment, or support in WHO-identified high-burden countries. Roles included non-governmental organisations (NGOs), global partnerships, or civil society representatives (37%), academic researchers and trainees (21%), health care workers (HCWs; 16%), TB-affected community members (15%), laboratory staff (6%), and Ministry of Health (MoH) representatives (5%). Participants worked across urban (74%), rural (41%), and peri-(semi)-urban (39%) settings, often spanning multiple environments (Table 1).

**Table 1.** Stakeholder demographics.

Characteristic	N (%)
Primary role (n = 274)	
NGOs, global partnerships, and civil society	101 (37%)
Academic researchers and trainees	58 (21%)
Health care workers	45 (16%)
TB-affected community members	40 (15%)
Laboratory staff	16 (6%)
Ministry of Health	14 (5%)
Setting(s) of care, treatment, or support of people with TB (n = 274)^A^	
Urban	204 (74%)
Rural	113 (41%)
Peri-(semi)urban	107 (39%)
Work experience in TB field (n = 239)	
Mean (SD), years	14.1 (9.0)
Median (IQR), years	12 (7–20)
Primary health facility for work or collaboration (n = 239)	
Publicly (government) funded clinic/hospital	109 (46%)
NGO	63 (26%)
Academic/research institutions	45 (19%)
Multisectoral health partnerships	10 (4%)
Community health centre	6 (3%)
Privately funded clinic/hospital	4 (2%)
Private practice	2 (1%)
TB care provision and populations served (n = 239)	
Both adult and paediatric	107 (45%)
Adult	41 (17%)
Paediatric	7 (3%)
Do not provide TB care in professional role^B^	84 (35%)

IQR = interquartile range; n = number; NGO = non-governmental organisation.

A Total surpasses 100% as respondents may be involved in TB support in multiple settings.

B Includes respondents professionally engaged in TB work whose roles involve programmatic, administrative, or research activities rather than direct patient care.

Among 239 respondents professionally engaged in TB work, median experience was 12 years (interquartile range [IQR]: 7–20). Forty-five percent provided care for both adults and children, 17% adults only, and 3% children only, while 35% did not provide TB care in their professional role.

Of 155 respondents professionally providing TB care, 81% reported facility capacity to diagnose multidrug-resistant TB (MDR-TB); 40% of these were MDR-TB referral centres, while 19% lacked in-house MDR-TB diagnostic capacity and referred all suspected cases externally. Among 152 respondents, 42% reported unreliable electricity (scheduled outages 27%; inconsistent power 10%; off-grid 6%).

### Stakeholder preferences for TB POC diagnostic features, barriers to testing, and thresholds for timely and affordable TB diagnosis

**Figure 1.**
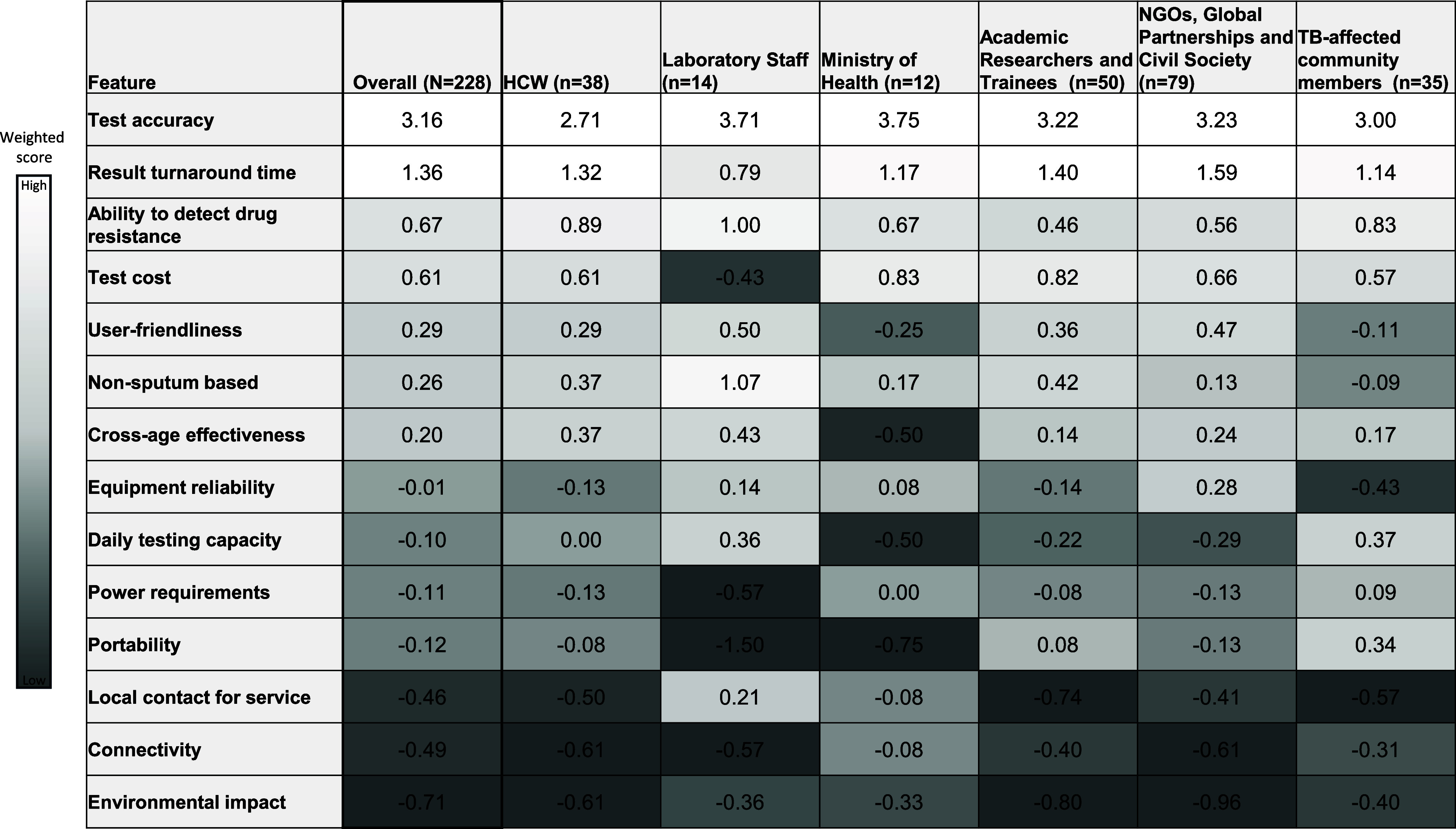
Weighted scores for TB point-of-care diagnostic features, overall and by role. HCW = health care worker; NGOs = non-governmental organisations; n = number of respondents.

Test accuracy was the most critical feature, receiving the highest overall score (3.16 on a −3 to +4 scale) and the top feature across all stakeholder groups. Result turnaround time was the second most valued feature (1.36), though laboratory staff rated it lower (0.79) compared to other groups (Figure 1). Subgroup analyses confirmed these top priorities across TB burden settings and TB evaluation responsibility (Supplementary Data Figures S1 and S2).

Drug-resistance detection was valued by clinical and patient-facing stakeholders, including laboratory staff (1.00), HCWs (0.89), and TB-affected community members (0.83). In contrast, academic researchers and NGOs assigned it moderate scores (0.46 and 0.56, respectively). Test cost was broadly valued, with MoH representatives and academic researchers assigning higher scores than other groups (0.83 and 0.82, respectively). However, laboratory staff deprioritised this feature (−0.43).

Mid-priority features such as user-friendliness, non-sputum-based tests, cross-age effectiveness, equipment reliability, daily testing capacity, power requirements, and portability were moderately valued overall but varied in importance across stakeholder groups. Local service providers, connectivity, and environmental impact were deprioritised by most groups.

**Figure 2.**
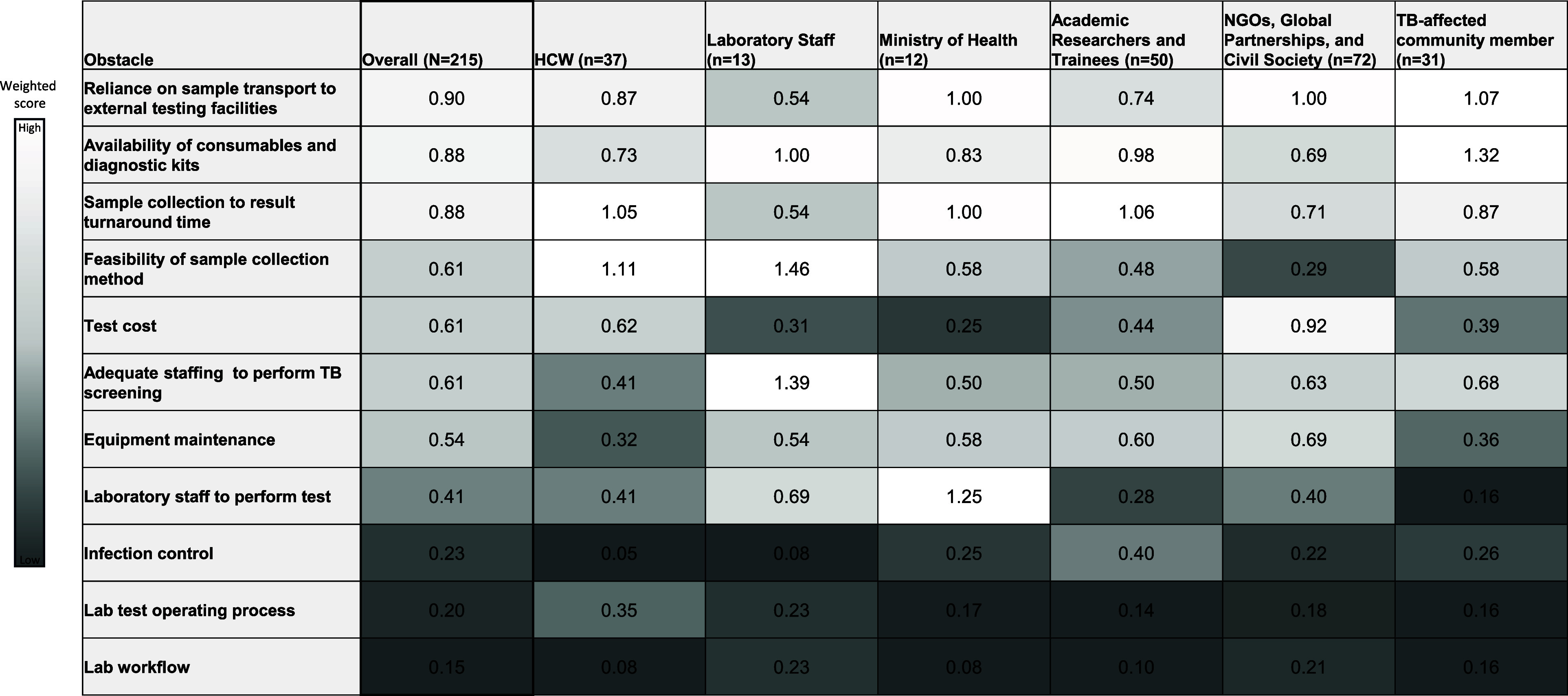
Weighted scores for obstacles to TB testing, overall and by role. HCW = health care worker; NGOs = non-governmental organisations; n = number of respondents.

Among 215 respondents, the greatest obstacles to TB testing were reliance on sample transport to external testing facilities (0.90), availability of consumables and diagnostic kits (0.88), and delays from sample collection to result turnaround (0.88) (Figure 2).

Feasibility of sample collection method was more role-dependent, rated higher by laboratory staff (1.46) and HCWs (1.11) versus NGOs (0.29). Laboratory staff identified inadequate staffing to perform TB screening as a key barrier (1.39), while MoH representatives assigned highest concern to availability of laboratory personnel to conduct TB tests (1.25). Despite role-specific concerns, the overall score for laboratory staffing was comparatively lower (0.41).

Test cost received a moderate score (Overall: 0.61) but was rated more strongly by NGO and civil society respondents (0.92), while laboratory staff (0.31) and MoH respondents (0.25) assigned it lower priority. Laboratory workflow (0.15), test operating processes (0.20), and infection control (0.23) scored among the lowest across stakeholder groups.

**Figure 3.**
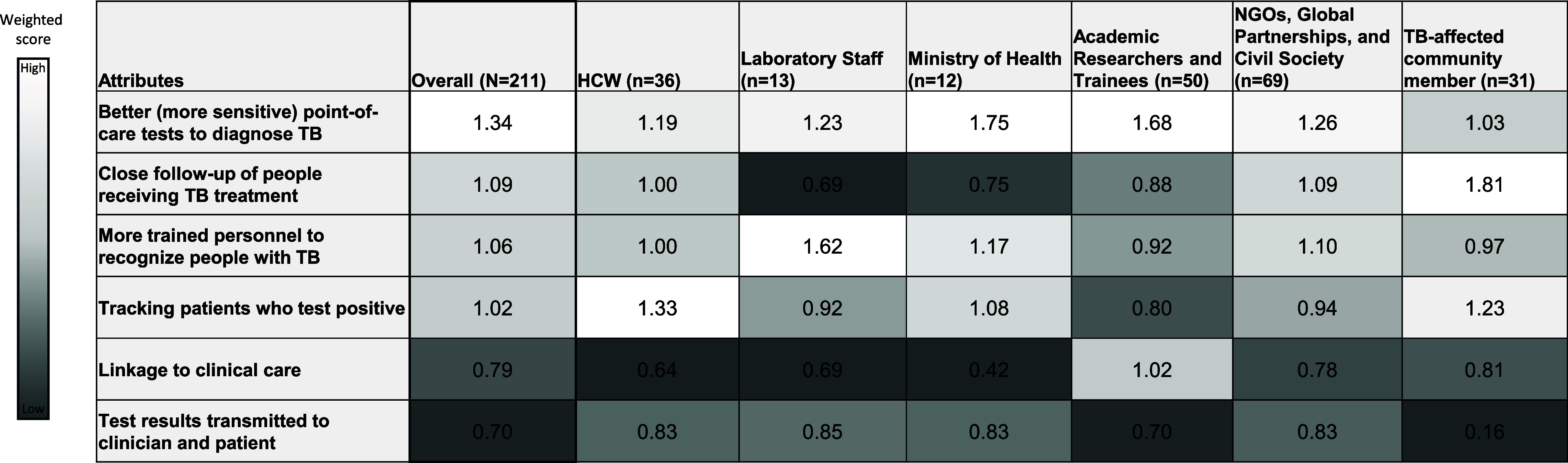
Weighted scores for attributes to TB response in local setting, overall and by role. HCW = health care worker; NGOs = non-governmental organisations; n = number of respondents.

Among 211 respondents, highest rated attributes for strengthening TB response were availability of better (more sensitive) POC diagnostics (1.34), with strongest support from MoH respondents (1.75), academic researchers and trainees (1.68), and laboratory staff (1.23) (Figure 3). Close follow-up of individuals receiving TB treatment ranked second (1.09), valued most by TB-affected community members (1.81), who also rated tracking of people who test positive highly (1.23), consistent with HCWs (1.33) and MoH representatives (1.08). More trained personnel to recognise TB also scored strongly (Overall: 1.06), particularly among laboratory staff (1.62) and MoH respondents (1.17). Linkage to clinical care (0.79) and test result transmission to clinicians and people with TB (0.70) received lowest scores overall.

Overall (N = 213) median maximum acceptable wait time for TB diagnostic results was 2 h (IQR: 1–24), consistent across TB evaluation responsibilities and burden settings. Laboratory staff reported the shortest median (1.5 h), while MoH representatives reported the longest (24 h); all other roles reported 2 h (Table 2).

**Table 2.** Maximum acceptable TB diagnostic result wait time and cost.

Category/Group	Outcome	Median (IQR)	n
Overall	Wait time (h)	2 (1–24)	213
	Cost (US$)	5 (2–10)	220
TB evaluation responsibilities			
Evaluate for TB	Wait time (h)	2 (1–12)	107
	Cost (US$)	5 (2–10)	110
Do not evaluate for TB	Wait time (h)	2 (0.5–24)	106
	Cost (US$)	5 (2–6)	110
Setting of TB care provision			
High-TB-burden countries	Wait time (h)	2 (0.75–24)	190
	Cost (US$)	5 (2–10)	197
Low-TB-burden countries	Wait time (h)	2 (1–24)	23
	Cost (US$)	7 (5–20)	23
Primary role			
Health care worker	Wait time (h)	2 (1–12)	38
	Cost (US$)	8 (2–10)	38
Laboratory staff	Wait time (h)	1.5 (1–2.5)	12
	Cost (US$)	10 (1.5–20)	13
Ministry of Health	Wait time (h)	24 (0.5–24)	11
	Cost (US$)	2.5 (2–6)	12
Academic researchers and trainees	Wait time (h)	2 (1–8)	49
	Cost (US$)	5 (5–10)	50
NGOs, global partnerships, and civil society	Wait time (h)	2 (0.5–12)	73
	Cost (US$)	5 (2–5)	75
TB-affected community member	Wait time (h)	2 (0.5–24)	30
	Cost (US$)	2.5 (1–5)	32

Median values are reported with interquartile ranges (IQRs). Wait time is expressed in hours (h), cost in US dollars (US$). Sample sizes (n) vary across groups due to voluntary survey completion.

NGO = non-governmental organisation.

Overall median maximum acceptable cost was US$5 (IQR: 2–10), consistent across TB evaluation responsibilities, although respondents from low-burden countries reported a higher median (US$7; IQR: 5–20). Laboratory staff reported the highest (US$10; IQR: 1.5–20), followed by HCWs (US$8; IQR: 2–10). MoH representatives and TB-affected community members reported the lowest (US$2.5; IQR: 2–6 and 1–5, respectively). Academic researchers and NGOs both reported US$5.

### Health care worker training and perceived TB screening coverage

Median percentage of HCWs in respondents’ local settings who were adequately trained to recognise TB cases was 50% (IQR: 20%–70%). Median percentage of individuals appropriately screened was 60% (IQR: 40%–80%) – see Supplementary Data Table S1. Respondents reported HCWs received adult TB screening training yearly (36%) or semi-annually (23%), while nearly one third reported rare or no training and 9% reported more frequent sessions. Paediatric TB screening training was less frequent: over half of the respondents reported rare or no training (53%).

## DISCUSSION

Accuracy and result turnaround time were universal priorities, reflecting the fundamental goal to promptly identify and treat TB.^6,12^ Similar preferences in South Africa, India, and other low-resource settings showed that accuracy and immediacy were drivers of TB rapid test adoption.^13,14^ Although laboratory staff placed lower importance on turnaround time, likely reflecting sample-processing constraints and emphasis on analytical quality,^15–17^ they reported the shortest acceptable wait time (1.5 h) and MoH representatives the longest (24 h). Differences may reflect operational versus policy roles and varied turnaround time definitions.^15,18,19^ These findings align with WHO TPP guidance calling for results within a single encounter to minimise pre-treatment loss and enable same-day clinical decision-making.^2^

Drug-resistance detection ranked highly, particularly among HCWs, laboratory staff, and TB-affected community members, reflecting complexities of managing drug-resistant TB and lived experiences navigating prolonged diagnostic pathways and suboptimal treatment. Despite increasing demand, few POC tools offer this.^20^ WHO TPP identifies this as ideal for appropriate treatment initiation and reducing diagnostic drop-off.^2^ Our results highlight ongoing MDR-TB testing centralisation: although most providers reported diagnostic capacity, 19% lacked in-house testing and referred all cases externally, underscoring the opportunity for decentralised POC platforms with drug-resistance detection.

Respondents indicated clear performance thresholds, with median maximum acceptable turnaround time of 2 h and maximum acceptable test cost of US$5, aligning closely with WHO TPP guidance.^2^ Evidence-based pricing is essential for equitable uptake of new TB diagnostics.^2^ Cost was moderately prioritised overall but deprioritised by laboratory staff, while MoH and TB-affected community members reported the lowest acceptable cost (US$2.50), suggesting procurement-user disconnects and underscores affordability concerns. A discrete choice experiment among adults undergoing TB evaluation in high-burden countries identified patient preferences for universally affordable, accurate, and rapid testing.^21^ Our study extends this evidence by incorporating perspectives from patient and system-level stakeholders.

Preferences diverged around daily testing capacity, portability, and power requirements. Laboratory staff and TB-affected community members valued daily testing capacity, highlighting its importance for efficient workflows and reducing delays. Community members also prioritised portability and minimal power requirements. Conversely, laboratory staff deprioritised these features, reflecting orientation toward fixed, high-throughput platforms and concerns about quality control, workflow disruption, and preservation of their professional roles.^22–24^ Infrastructure barriers were prominent with 42% of respondents reporting unreliable electricity, and sample transport to external facilities, consumable stockouts, and delays from collection to result were the highest ranked obstacles. Unreliable electricity reinforces the need for reliable diagnostics with minimal power requirements, consistent with WHO TPP guidance calling for diagnostics usable in decentralised, low-resource settings, including battery-powered or near-POC platforms with sufficient throughput and affordability.^2,23^

Non-sputum tests were broadly supported, particularly by laboratory staff. Similar preferences have been documented in India, South Africa, and Vietnam citing faster workflows and lower infection risk.^16^ This is especially relevant for people living with HIV and children, who often cannot produce sputum and are among the most vulnerable populations.^25,26^

System-integration features including connectivity, local contact for service, and environmental impact scored lowest, suggesting performance and accessibility priorities over longer-term operational and sustainability concerns, though these remain important for implementation success at scale.

System-level barriers – sample transport, consumable stockouts, and collection-to-result delays – mirror well-documented bottlenecks in high-burden settings.^27–31^ Stakeholders broadly agreed on the importance of improved POC sensitivity, follow-up systems, and training to strengthen TB response. Training gaps in paediatric TB screening were evident: 53% of HCWs rarely or never received such training, echoing Zambian findings where training gaps contributed to poor knowledge and practices.^32^ More broadly, respondents estimated that only half of HCWs were adequately trained to recognise TB, underscoring workforce constraints beyond paediatrics. Training improves detection^33^ by enhancing symptom recognition, diagnostic use, and timely treatment.^14,34^ Addressing system-level barriers and scaling targeted, frequent paediatric TB screening training alongside diagnostic innovation is critical for effective implementation and improved outcomes for the most vulnerable populations.^35^

POC tools must be paired with training, reliable supply chains, streamlined sample transport, and linkages to care to achieve real-world impact. In Central Asia, political commitment and multisectoral collaboration enabled deployment of community-led digital health tools and primary health care–integrated diagnostics, improving diagnostic reach while addressing decentralised access challenges.^27^ A portfolio approach combining high-throughput platforms for centralised facilities and portable, infrastructure-independent devices for peripheral settings, where power and infrastructure challenges demand adaptable technologies, offers a pragmatic path forward. Together, these findings reinforce that stakeholders envision POC as not only faster and more accurate, but decentralised, affordable, and feasible within constrained infrastructure.

Significant input from TB-affected communities, NGOs, HCWs, and researchers strengthens generalisability. Subgroup analyses demonstrated similar priorities across TB burden settings and evaluation responsibilities, though consumable stockouts were deprioritised in low-burden settings, suggesting this barrier is more acute in high-burden settings. Such variation reinforces the value of a portfolio approach that tailors diagnostics to setting-specific challenges rather than a universal design.

This study has limitations. The sample, while diverse and global, was not population-based and may be subject to response bias. Structured ranking required simplification of diagnostic features, potentially limiting nuance. The survey was conducted in English, potentially limiting participation from non-English speakers. Imbalanced stakeholder representation may have influenced relative emphasis of preferences. Laboratory staff were relatively underrepresented (n = 16; 6%), potentially underestimating laboratory operational barriers essential for implementation and performance. Findings rely on self-reported perspectives rather than observed practice, and stated thresholds for turnaround time and cost may not fully reflect real-world adoption.

## CONCLUSION

This study highlights how stakeholder priorities converge and diverge, reinforcing that future TB diagnostic development must prioritise accuracy, speed, and affordability with solutions for system-level constraints and training to maximise uptake and translate advancements into real-world impact. Incorporating these perspectives into diagnostic development, policy, and implementation can help close the diagnostic gap and accelerate TB elimination. A portfolio approach is needed, combining high-throughput solutions for centralised facilities with adaptable, infrastructure-independent tools for peripheral settings to meet diverse needs.

## Supplementary Material
