# Supplementary material for: Stakeholder engagement to identify barriers to implementation and inform the development of point-of-care diagnostics for TB
Source: IJTLD Open. 2026 Apr 13;3(4):255–61. doi: 10.5588/ijtldopen.25.0680 (PMC13080304; doi:10.5588/ijtldopen.25.0680)
Supplement: Supplementary file 1 [file ijtldopen25-0680_supplementarydata1.pdf]

## Supplementary Data

### *Table S1. Healthcare worker training and perceived TB screening coverage*

| | |
|---|---|
| Percentage of Healthcare Workers in Local Setting Adequately Trained to Recognize TB Cases (n=207) | |
| Median (IQR) | 50 (20-70) |
| Percentage of Individuals in Local Setting Requiring TB Screening and Properly Screened (n=207) | |
| Median (IQR) | 60 (40-80) |
| Frequency of healthcare worker training on TB screening in adults (n=183) | N (%) |
| Weekly | 3 (2%) |
| Monthly | 13 (7%) |
| Semi-Annually | 43 (23%) |
| Yearly | 65 (36%) |
| Rarely | 53 (29%) |
| Never | 6 (3%) |
| Frequency of healthcare worker training on TB screening in children (n=183) | |
| Monthly | 10 (5%) |
| Semi-Annually | 28 (15%) |
| Yearly | 47 (26%) |
| Rarely | 88 (48%) |
| Never | 10 (5%) |

*IQR=interquartile range; n=number of respondents*

### *Figure S1. Weighted scores for TB POC diagnostic feature, overall and by TB burden setting*

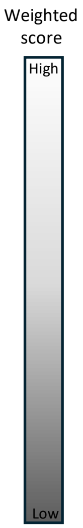


| Feature | Overall (N=228) | High TB burden countries (n=205) | Non-High TB burden countries (n=23) |
|---|---|---|---|
| Test accuracy | 3.16 | 3.18 | 3.04 |
| Result turnaround time | 1.36 | 1.32 | 1.74 |
| Ability to detect drug resistance | 0.67 | 0.62 | 1.09 |
| Test cost | 0.61 | 0.62 | 0.57 |
| User-friendliness | 0.29 | 0.32 | 0.00 |
| Non-sputum based | 0.26 | 0.20 | 0.74 |
| Cross-age effectiveness | 0.20 | 0.21 | 0.13 |
| Equipment reliability | -0.01 | 0.02 | -0.26 |
| Daily testing capacity | -0.10 | -0.09 | -0.13 |
| Power requirements | -0.11 | -0.05 | -0.61 |
| Portability | -0.12 | -0.11 | -0.22 |
| Local contact for service | -0.46 | -0.48 | -0.35 |
| Connectivity | -0.49 | -0.50 | -0.39 |
| Environmental impact | -0.71 | -0.73 | -0.57 |

*n=number of respondents*

***Figure S2. Weighted scores for TB POC diagnostic feature, overall and by TB evaluation responsibility***

Weighted score

High

Low

| Feature | Overall (N=228) | Evaluate for TB (n=116) | Do not evaluate for TB (n=112) |
|---|---|---|---|
| Test accuracy | 3.16 | 3.05 | 3.27 |
| Result turnaround time | 1.36 | 1.32 | 1.41 |
| Ability to detect drug resistance | 0.67 | 0.75 | 0.59 |
| Test cost | 0.61 | 0.63 | 0.60 |
| User-friendliness | 0.29 | 0.23 | 0.34 |
| Non-sputum based | 0.26 | 0.31 | 0.21 |
| Cross-age effectiveness | 0.20 | 0.22 | 0.18 |
| Equipment reliability | -0.01 | -0.07 | 0.05 |
| Daily testing capacity | -0.10 | -0.03 | -0.16 |
| Power requirements | -0.11 | -0.13 | -0.09 |
| Portability | -0.12 | -0.26 | 0.02 |
| Local contact for service | -0.46 | -0.48 | -0.45 |
| Connectivity | -0.49 | -0.53 | -0.45 |
| Environmental impact | -0.71 | -0.50 | -0.91 |

*n=number of respondents*

***Figure S3. Weighted scores for obstacles to TB testing, overall and by TB burden setting***

Weighted score

High

Low

| Obstacle | Overall (N=215) | High TB burden countries (n=193) | Non-High TB burden countries (n=22) |
|---|---|---|---|
| Reliance on sample transport to external testing facilities | 0.90 | 0.90 | 0.86 |
| Availability of consumables and diagnostic kits | 0.88 | 0.96 | 0.18 |
| Sample collection to result turnaround time | 0.88 | 0.86 | 1.00 |
| Feasibility of sample collection method | 0.61 | 0.61 | 0.59 |
| Test cost | 0.61 | 0.60 | 0.64 |
| Adequate staffing to perform TB screening | 0.61 | 0.61 | 0.54 |
| Equipment maintenance | 0.54 | 0.54 | 0.59 |
| Laboratory staff to perform test | 0.41 | 0.38 | 0.59 |
| Infection control | 0.23 | 0.24 | 0.14 |
| Lab test operating process | 0.20 | 0.18 | 0.41 |
| Lab workflow | 0.15 | 0.11 | 0.45 |

*n=number of respondents*

***Figure S4. Weighted scores for obstacles to TB testing by TB evaluation responsibility***

Weighted score

High

Low

| Obstacle | Overall (N=215) | Evaluate for TB (n=108) | Do not evaluate for TB (n=107) |
|---|---|---|---|
| Reliance on sample transport to external testing facilities | 0.90 | 0.85 | 0.94 |
| Availability of consumables and diagnostic kits | 0.88 | 0.84 | 0.92 |
| Sample collection to result turnaround time | 0.88 | 0.94 | 0.81 |
| Feasibility of sample collection method | 0.61 | 0.63 | 0.58 |
| Test cost | 0.61 | 0.65 | 0.56 |
| Adequate staffing to perform TB screening | 0.61 | 0.61 | 0.59 |
| Equipment maintenance | 0.54 | 0.51 | 0.58 |
| Laboratory staff to perform test | 0.41 | 0.44 | 0.36 |
| Infection control | 0.23 | 0.15 | 0.32 |
| Lab test operating process | 0.20 | 0.20 | 0.20 |
| Lab workflow | 0.15 | 0.17 | 0.13 |

*n=number of respondents*

***Figure S5. Weighted scores for attributes to TB response in local setting, overall and by TB burden setting***

Weighted score

High

Low

| Attributes | Overall (N=211) | High TB burden countries (n=189) | Non-High TB burden countries (n=22) |
|---|---|---|---|
| Better (more sensitive) point-of-care tests to diagnose TB | 1.34 | 1.31 | 1.64 |
| Close follow-up of people receiving TB treatment | 1.09 | 1.09 | 1.04 |
| More trained personnel to recognize people with TB | 1.06 | 1.03 | 1.32 |
| Tracking patients who test positive | 1.02 | 1.05 | 0.77 |
| Linkage to clinical care | 0.79 | 0.79 | 0.82 |
| Test results transmitted to clinician and patient | 0.70 | 0.73 | 0.41 |

*n=number of respondents*

***Figure S6. Weighted scores for attributes to TB response in local setting, overall and by TB evaluation responsibility***

Weighted score

High

Low

| Attributes | Overall (N=211) | Evaluate for TB (n=107) | Do not evaluate for TB (n=104) |
|---|---|---|---|
| Better (more sensitive) point-of-care tests to diagnose TB | 1.34 | 1.28 | 1.40 |
| Close follow-up of people receiving TB treatment | 1.09 | 0.94 | 1.23 |
| More trained personnel to recognize people with TB | 1.06 | 1.23 | 0.88 |
| Tracking patients who test positive | 1.02 | 1.03 | 1.02 |
| Linkage to clinical care | 0.79 | 0.79 | 0.79 |
| Test results transmitted to clinician and patient | 0.70 | 0.72 | 0.68 |

*n=number of respondents*

***Table S7. Proportion of respondents by country of care, treatment, or support of people with TB (N=274)***

| Country | n (%) | Country | n (%) |
|---|---|---|---|
| Afghanistan | 8 (2.9) | Libya | 2 (0.7) |
| Albania | 1 (0.4) | Liechtenstein | 1 (0.4) |
| Algeria | 1 (0.4) | Lithuania | 1 (0.4) |
| Andorra | 1 (0.4) | Luxembourg | 1 (0.4) |
| Angola | 3 (1.1) | Madagascar | 3 (1.1) |
| Antigua and Barbuda | 1 (0.4) | Malawi | 12 (4.4) |
| Argentina | 2 (0.7) | Malaysia | 2 (0.7) |
| Armenia | 2 (0.7) | Maldives | 1 (0.4) |
| Australia | 1 (0.4) | Mali | 1 (0.4) |
| Austria | 1 (0.4) | Malta | 1 (0.4) |
| Azerbaijan | 1 (0.4) | Marshall Islands | 1 (0.4) |
| Bahamas | 1 (0.4) | Mauritania | 1 (0.4) |
| Bahrain | 1 (0.4) | Mauritius | 1 (0.4) |
| Bangladesh | 4 (1.5) | Mexico | 4 (1.5) |
| Barbados | 1 (0.4) | Micronesia | 1 (0.4) |
| Belarus | 5 (1.8) | Monaco | 1 (0.4) |
| Belgium | 2 (0.7) | Mongolia | 3 (1.1) |
| Belize | 1 (0.4) | Montenegro | 1 (0.4) |
| Benin | 1 (0.4) | Morocco | 1 (0.4) |
| Bhutan | 1 (0.4) | Mozambique | 16 (5.8) |
| Bolivia | 4 (1.5) | Myanmar | 4 (1.5) |
| Bosnia and Herzegovina | 1 (0.4) | Namibia | 4 (1.5) |
| Botswana | 4 (1.5) | Nauru | 1 (0.4) |
| Brazil | 11 (4.0) | Nepal | 3 (1.1) |
| Brunei | 1 (0.4) | Netherlands | 1 (0.4) |
| Bulgaria | 1 (0.4) | New Zealand | 2 (0.7) |

| | | | |
|---|---|---|---|
| Burkina Faso | 1 (0.4) | Nicaragua | 1 (0.4) |
| Burundi | 2 (0.7) | Niger | 2 (0.7) |
| Cambodia | 9 (3.3) | Nigeria | 8 (2.9) |
| Cameroon | 5 (1.8) | North Korea | 2 (0.7) |
| Canada | 1 (0.4) | Norway | 1 (0.4) |
| Cabo Verde | 1 (0.4) | Oman | 1 (0.4) |
| Central African Republic | 1 (0.4) | Pakistan | 5 (1.8) |
| Chad | 2 (0.7) | Palau | 1 (0.4) |
| Chile | 2 (0.7) | Panama | 2 (0.7) |
| China | 1 (0.4) | Papua New Guinea | 2 (0.7) |
| Colombia | 2 (0.7) | Paraguay | 3 (1.1) |
| Comoros | 1 (0.4) | Peru | 6 (2.2) |
| Congo | 2 (0.7) | Philippines | 9 (3.3) |
| Costa Rica | 1 (0.4) | Poland | 1 (0.4) |
| Côte d'Ivoire | 2 (0.7) | Portugal | 1 (0.4) |
| Croatia | 1 (0.4) | Qatar | 1 (0.4) |
| Cuba | 2 (0.7) | Moldova | 2 (0.7) |
| Cyprus | 1 (0.4) | Romania | 1 (0.4) |
| Czech Republic | 1 (0.4) | Russia | 6 (2.2) |
| Democratic Republic of the Congo | 7 (2.6) | Rwanda | 2 (0.7) |
| Denmark | 1 (0.4) | Saint Kitts and Nevis | 1 (0.4) |
| Djibouti | 2 (0.7) | Saint Lucia | 1 (0.4) |
| Dominica | 1 (0.4) | Saint Vincent and the Grenadines | 1 (0.4) |
| Dominican Republic | 2 (0.7) | Samoa | 1 (0.4) |
| Ecuador | 3 (1.1) | San Marino | 1 (0.4) |
| Egypt | 1 (0.4) | Sao Tome and Principe | 1 (0.4) |
| El Salvador | 2 (0.7) | Saudi Arabia | 1 (0.4) |
| Equatorial Guinea | 1 (0.4) | Senegal | 2 (0.7) |
| Eritrea | 2 (0.7) | Serbia | 1 (0.4) |
| Estonia | 1 (0.4) | Seychelles | 2 (0.7) |
| Eswatini | 5 (1.8) | Sierra Leone | 3 (1.1) |
| Ethiopia | 10 (3.6) | Singapore | 1 (0.4) |
| Fiji | 1 (0.4) | Slovakia | 1 (0.4) |
| Finland | 1 (0.4) | Slovenia | 1 (0.4) |
| France | 1 (0.4) | Solomon Islands | 1 (0.4) |
| Gabon | 1 (0.4) | Somalia | 2 (0.7) |
| Gambia | 2 (0.7) | South Africa | 22 (8.0) |
| Georgia | 4 (1.5) | South Korea | 1 (0.4) |
| Germany | 2 (0.7) | Spain | 2 (0.7) |
| Ghana | 1 (0.4) | Sri Lanka | 1 (0.4) |
| Greece | 1 (0.4) | Sudan | 3 (1.1) |
| Grenada | 1 (0.4) | Suriname | 1 (0.4) |
| Guatemala | 5 (1.8) | Sweden | 1 (0.4) |
| Guinea | 1 (0.4) | Switzerland | 1 (0.4) |
| Guinea Bissau | 2 (0.7) | Syria | 1 (0.4) |
| Guyana | 1 (0.4) | Tajikistan | 9 (3.3) |
| Haiti | 1 (0.4) | Thailand | 3 (1.1) |
| Honduras | 2 (0.7) | North Macedonia | 1 (0.4) |
| Hong Kong | 1 (0.4) | Timor Leste | 1 (0.4) |

| | | | |
|---|---|---|---|
| Hungary | 1 (0.4) | Togo | 1 (0.4) |
| Iceland | 1 (0.4) | Tonga | 1 (0.4) |
| India | 31 (11.3) | Trinidad and Tobago | 1 (0.4) |
| Indonesia | 9 (3.3) | Tunisia | 1 (0.4) |
| Iran | 1 (0.4) | Turkey | 1 (0.4) |
| Iraq | 1 (0.4) | Turkmenistan | 1 (0.4) |
| Ireland | 1 (0.4) | Tuvalu | 1 (0.4) |
| Israel | 1 (0.4) | Uganda | 39 (14.2) |
| Italy | 1 (0.4) | Ukraine | 16 (5.8) |
| Jamaica | 1 (0.4) | United Arab Emirates | 1 (0.4) |
| Japan | 1 (0.4) | United Kingdom | 2 (0.7) |
| Jordan | 1 (0.4) | Tanzania | 8 (2.9) |
| Kazakhstan | 5 (1.8) | United States of America | 6 (2.2) |
| Kenya | 38 (13.9) | Uruguay | 1 (0.4) |
| Kiribati | 1 (0.4) | Uzbekistan | 6 (2.2) |
| Kuwait | 1 (0.4) | Vanuatu | 1 (0.4) |
| Kyrgyzstan | 13 (4.7) | Venezuela | 1 (0.4) |
| Laos | 3 (1.1) | Vietnam | 7 (2.6) |
| Latvia | 1 (0.4) | Yemen | 1 (0.4) |
| Lebanon | 2 (0.7) | Zambia | 17 (6.2) |
| Lesotho | 7 (2.6) | Zimbabwe | 22 (8.0) |
| Liberia | 6 (2.2) | | |

*Percentages reflect the proportion of respondents. Total exceeds 100% as respondents may be involved in TB care, treatment, or support in multiple countries.*
